# Cell-Based Therapies for Stroke: Are We There Yet?

**DOI:** 10.3389/fneur.2019.00656

**Published:** 2019-06-25

**Authors:** Mirja Krause, Thanh G. Phan, Henry Ma, Christopher G. Sobey, Rebecca Lim

**Affiliations:** ^1^The Ritchie Centre, Hudson Institute of Medical Research, Melbourne, VIC, Australia; ^2^Department of Obstetrics and Gynaecology, Monash University, Melbourne, VIC, Australia; ^3^Department of Medicine, Monash University, Melbourne, VIC, Australia; ^4^Department of Physiology, Anatomy and Microbiology, La Trobe University, Melbourne, VIC, Australia; ^5^Australian Regenerative Medicine Institute, Monash University, Melbourne, VIC, Australia

**Keywords:** stroke, clinical trial, stem cells, cell therapy, clinical trial costs

## Abstract

Stroke is the second leading cause of death and physical disability, with a global lifetime incidence rate of 1 in 6. Currently, the only FDA approved treatment for ischemic stroke is the administration of tissue plasminogen activator (tPA). Stem cell clinical trials for stroke have been underway for close to two decades, with data suggesting that cell therapies are safe, feasible, and potentially efficacious. However, clinical trials for stroke account for <1% of all stem cell trials. Nevertheless, the resources devoted to clinical research to identify new treatments for stroke is still significant (53–64 million US$, Phase 1–4). Notably, a quarter of cell therapy clinical trials for stroke have been withdrawn (15.2%) or terminated (6.8%) to date. This review discusses the bottlenecks in delivering a successful cell therapy for stroke, and the cost-to-benefit ratio necessary to justify these expensive trials. Further, this review will critically assess the currently available data from completed stroke trials, the importance of standardization in outcome reporting, and the role of industry-led research in the development of cell therapies for stroke.

## Introduction

### Background

Stroke has a devastating effect on the society worldwide. In addition to its significant mortality rate of 50% as reported in 5-year survival studies (1), it affects as many as 1 in 6 people in their lifetimes, and is the leading cause of disability worldwide (2). A stroke results in a complex interplay of inflammation and repair with effects on neural, vascular, and connective tissue in and around the affected areas of the brain (3). Therefore, sequelae of stroke such as paralysis, chronic pain, and seizures can persist long term and prevent the patient from fully reintegrating into society. Stroke therefore remains the costliest healthcare burden as a whole (4). In 2012, the total cost of stroke in Australia was estimated to be about $5 billion with direct health care costs attributing to $881 million of the total (5).

Unfortunately, treatment options for stroke are still greatly limited. Intravenous recombinant tissue plasminogen activator (tPA) and endovascular thrombectomy (EVT) are currently the only effective treatments available for acute stroke. However, there is only a brief window of opportunity where they can be successfully applied. EVT is performed until up to 24 h of stroke onset (6), while tPA is applied within 4.5 h of stroke onset. Notably, the recent WAKE-UP (NCT01525290) (7) and EXTEND (NCT01580839) trials have shown that this therapeutic window can be safely extended to 9 h from stroke onset. Furthermore, advancements in acute stroke care and neurorehabilitation have shown to be effective in improving neurological function (8). However, there are no treatments that offer restoration of function and as a result, many patients are left with residual deficits following a stroke. Cell-based therapies have shown promising results in animal models addressing the recovery phase following stroke (9). This is encouraging as currently, there are no approved treatment options addressing the reversal of neurological damages once a stroke has occurred (10).

The majority of data from animal studies and clinical trials demonstrate the therapeutic potential of stem cells in the restoration of central nervous system (CNS) function (11, 12), applicable to neurodegenerative diseases as well as traumatic brain injury. Transplanted stem cells were reportedly able to differentiate into neurons and glial cells, whilst supporting neural reconstruction and angiogenesis in the ischemic region of the brain (13). Previous work demonstrated the ability of mesenchymal stem cells (MSCs) to differentiate into neurons, astrocytes (14), endothelial cells (15, 16), and oligodendrocyte lineage cells (17) such as NG2-positive cells (18) *in vitro*, and undergo neuronal or glial differentiation *in vivo* (19). Bone marrow-derived mesenchymal stem cells (BMSCs) have shown potential to differentiate into endothelial cells *in vitro* (20). Additionally, both BMSCs and adipose stem cells (ASCs) have been shown to demonstrate neural lineage differentiation potential *in vitro* (21–23). Furthermore, stem cells are able to modulate multiple cell signaling pathways involved in endogenous neurogenesis, angiogenesis, immune modulation and neural plasticity, sometimes in addition to cell replacement (3). The delivery of stem cells from the brain, bone marrow, umbilical cord, and adipose tissue, have been reported to reduce infarct size and improve functional outcomes regardless of tissue source (9). While these were initially exciting reports, they raise the question as to the validity of the findings to date since these preclinical reports are almost uniformly positive. The absence of scientific skepticism and robust debate may in fact have negated progress in this field.

Cell-based therapies have been investigated as a clinical option since the 1990s. The first pilot stroke studies in 2005 investigated the safety of intracranial delivery of stem cells (including porcine neural stem cells) to patients with chronic basal ganglia infarcts or subcortical motor strokes (24, 25). However, since the publication of these reports, hundreds of preclinical studies have shown that a variety of cell types including those derived from non-neural tissues can enhance structural and functional recovery in stroke. Cell therapy trials, mainly targeted at small cohorts of patients with chronic stroke, completed in the 2000s, showed satisfactory safety profiles and suggestions of efficacy (10). Current treatments such as tPA and EVT only have a narrow therapeutic window, limited efficacy in severe stroke and may be accompanied by severe side effects. Specifically, the side effects of EVT include intracranial hemorrhage, vessel dissection, emboli to new vascular territories, and vasospasm (26). The benefit of tPA for patients with a severe stroke with a large artery occlusion can vary significantly (27). This is mainly due to the failure (<30%) of early recanalisation of the occlusion. Thus, despite the treatment options stroke is still a major cause of mortality and morbidity, and there is need for new and improved therapies.

Stem cells have been postulated to significantly extend the period of intervention and target subacute as well as the chronic phase of stroke. Numerous neurological disorders such as Parkinson's disease (12, 28), Alzheimer's disease (29), age-related macular degeneration (30), traumatic brain injury (31), and malignant gliomas (32) have been investigated for the applicability of stem cell therapy. These studies have partly influenced the investigation of stem cell therapies for stroke. A small fraction of stem cell research has been successfully translated to clinical trials. As detailed in Table 1, most currently active trials use neuronal stem cells (NSCs), MSCs or BMSCs (35–37), including conditionally immortalized neural stem-cell line (CTX-DP) CTX0E03 (38), neural stem/progenitor cells (NSCs/NPSCs) (e.g., NCT03296618), umbilical cord blood (CoBis2, NCT03004976), adipose (NCT02813512), or amnion epithelial cells (hAECs, ACTRN 1261800076279) (39).

**Table 1:** Challenges and bottlenecks of stem cell therapy and clinical trials using stem cells (33, 34).

**Challenges/Bottlenecks**	
**TRIAL RELATED CHALLENGES**	
High costs	- *Increases with each trial phase*
Lengthy timelines	- *Average of 3.5–4 years per trial phase (estimated from data listed in Table 2)*
Difficulties in recruitment and retention of patients	- *Especially for acute stroke with a treatment time frame of <24 h; increased drop-out rates for longterm follow-up (>1 year after treatment)*
Insufficiencies in clinical research workforce (lack of specific training)	- *Disconnect between clinical research and medical care*
Strict regulations and admin barriers	- *Variations between different regulatory bodies; Designing a trial to answer a scientific question while considering the well-being of the patient and also adhere to regulations*
Complexity/Difficulties in maintaining and monitoring safety	- *Unexperienced personnel due to lack of specific training;*
Data collection and interpretation	- *Missing standardization*
Missing standardization of outcome reporting	- *Leads to biases; Data from several trials cannot be analyzed and compared*
**TREATMENT AND THERAPY RELATED CHALLENGES**	
Limited source of stem cells	- *Decrease in number/function of BMSCs in aged persons* - *Allogeneic MSCs need to be passaged to cover demand*
Optimal time frame for treatment	- *Decrease SC tropism toward brain with time* - *Mechanistic targets for cell therapy differ depending on time point of treatment* - *Insufficient cell amounts if autologous samples are used (depending on cell type)*
Limitations of SCs	- *Low yield* - *Heterogenous populations with difference in potential and efficacy*
Limitation in production processes	- *Lack of enabling technologies for cell-therapy bioprocessing at scale*
Adverse effects	- *Tumor formation; Immune rejection; Cells trapped in brain vessels or lung*

## Bottlenecks and Challenges of Cell Therapies

The development of a cell therapy for stroke is challenging for a number of reasons and these are detailed in Table 1. Each cell type requires testing for safety and efficacy to mitigate risks such as tumor formation. Identifying the ideal cell type for stroke has been hampered by the lack of data around clinical efficacy, as well as by the complex logistics and ethical concerns. The latter being a great hurdle for the use of fetal and embryonic stem cells in particular. The mechanisms of action of each cell type (i.e., cell replacement, growth factor secretion, and/or sequestration of inflammation) must be considered when choosing the appropriate route and timing of administration as these can directly influence treatment efficacy.

A critical translational consideration for stroke is the identification of the optimal route of administration. Different routes have been used in animal models for the transplantation of stem cells, including intracerebral (40), intracranial (41, 42), intranasal (43) or via stereotaxic infusion (44), and it is worth noting that all of these studies reported improvement in functional outcomes. Several different routes have been used in clinical trials (see Table 2); where the most common routes are intravenous infusion and intracerebral transplant. While different routes of administration have been compared in several reviews (58, 59), the optimal route has yet to be defined. Nevertheless, there is no evidence that a specific route of administration has significant effect on clinical efficacy (60–63).

**Table 2:** Completed and active clinical trials using cell therapy to treat stroke.

	**Trial number**	**Trial name**	**Current status**	**Study start and end date**	**Duration [y]**	**Phase**	**Sponsor**	**No. of participants**	**Cell type**	**Cell dose**	**Route**	**Time from stroke onset**	**References**
**COMPLETED**													
1	NCT00152113		Compl	2005–2008	3	1	Research Hospital	5	Hematopoietic Stem Cell	5 × 10^6^ CD34+/kg, 1 × 10^6^ CD3+/kg	IV	Prophylactic	(45)
2	NCT00473057		Compl	2005–2011	6	1	University/College	12	Autologous BMSCs	500 million	IA	90 days	
3	NCT00535197		Compl	2007–2012	5	1,2	University/College	5	Autologous BMSCs	Max. 1 × 10^8^	IA	<7 days	(46)
4	NCT01501773		Compl	2008–2011	3	2	Industry	120	Autologous BMSCs	30–500 million	IV	7–30 days	(44, 47)
5	NCT00761982		Compl	2008–2011	3	1,2	University Hospital	20	Autologous BMSCs	1.59 × 10^8^	IA	5–9 days	(48)
6	NCT00950521		Compl	2009–2010	1	2	University Hospital	30	Hematopoietic CD34+ stem cells	2–8 million	ICb	6–60 months	(49)
7	NCT02425670	InVeSt	Compl	2009–2010	1	2	Research Institute	120	Autologous BMSCs	30–500 million	IV	7–29 days	(47)
8	NCT00859014		Compl	2009–2013	4	1	University/College	25	Autologous BMSCs	10 × 10^6^/kg	IV	24–72 h	(50)
9	NCT00875654	ISIS-HERMES	Compl	2010–2017	7	2	Hospital	31	Autologous mesenchymal stem cells	100 or 300 million	IV	<6 weeks	
10	NCT01287936	SanBio	Compl	2011–2015	4	1,2	Industry	18	Autologous modified Stromal Cells (SB623)	2.5, 5.0, or 10 million	IC	6–60 months	(51)
11	NCT01436487	MASTERS-1	Compl	2011–2015	4	2	Industry	134	Allogenic BMSCs (MultiStem)	400 or 1200 million	IV	24–48 h	(52, 53)
12	NCT01297413		Compl	2011–2017	6	1,2	Research Institute	20	Allogenic BMSCs	0.5–1.5 × 10^6^/kg	IV	>6 months	
13	NCT02117635	PISCES-II	Compl	2014–2016	2	2a	Industry	23	Allogenic NSCs (CTX DP)	20 million cells	ICb	<4 weeks	(38)
14	NCT01678534	AMASCIS-01	Compl	2014–2018	4	2	University Hospital	19	Allogenic ADSCs	1 million units/kg	IV	<2 weeks	(54)
15	NCT03080571		Compl	2015–2016	1	1	Industry	38	Autologous intra-arterial BM-MNCs	–	IA	0–15 days	
16	NCT02397018	CoBIS1	Compl	2015–2017	2	1	Research Institute	124	Allogeneic umbilical cord blood stem cells	0.5–5 × 10^7^/kg	IV	3–10 days	(55)
17	NCT02813512		Compl	2017–2018	1	1	Industry	3	Autologous ADSCs	–	ICb	>6 months	
**ACTIVE**													
1	NCT01151124	PISCES	Not recr	2010–2023	13	1,2	Industry	12	Allogenic NSCs (CTX DP)	2, 5, 10, or 20 million	ICb	6 months to 5 years	(38)
2	NCT01716481	STARTING-2	Recr	2012–2017	5	3	Industry	60	Autologous MSCs	1 × 10^6^cells/kg	IV	<90 days	(56)
3	NCT03296618		Not recr	2012–2018	6	1	Industry	18	NSCs (NSI-566)	1.2 × 10^7^-8 × 10^7^	IC	3–24 months	
4	NCT02178657	IBIS	Recr	2014–2018	4	2	Research Institute	76	Autologous bone marrow mononuclear cells	2 or 5 × 10^6^/kg	IA	1–7 days	(57)
5	NCT02448641	ACTIsSIMA	Not recr	2016–2019	3	2	Industry	156	Autologous modified Stromal Cells (SB623)	2.5 or 5 million	IC	6–90 months	
6	NCT02795052	NEST	Recr	2016–2020	4	n.d.	Industry	300	Autologous BMSCs	–	IV, IN	>6 months	
7	NCT03371329		Recr	2017–2018	1	1	Hospital	12	Allogenic BMSCs	0.5, 1, 2 × 10^6^/kg	IV, IT	<72 h	
8	NCT03004976	CoBIS2	Recr	2017–2019	2	2	Research Institute	100	Allogeneic umbilical cord blood infusion	0.5–5 × 10^7^/kg	IV	3–10 days	
9	NCT03629275	PISCESIII	Recr	2018–2019	1	2b	Industry	110	Allogenic NSCs (CTX0E03)	20 million	ICb	6–12 months	
10	NCT03545607	MASTERS-2	Recr	2018–2020	2	3	Industry	300	Allogenic BMSCs (MultiStem)	1.2 billion	IV	24–36 h	(53)
11	NCT03570450	RESSTORE-1	Recr	2018–2020	2	1	University Hospital	15	ADSCs	1.1, 2.1, 2.5, 3.1 × 10^6^/kg	IV	24–48 h	
12	NCT02961504	TREASURE	Recr	2017–2020	3	2,3	Industry	220	Allogenic BMSCs, HLCM051 (MultiStem)	1.2 billion	IV	18–36 h	
13	ACTRN12618000076279	I-ACT	Recr	2019–2021	3	1	Research Institute	15	Allogenic hAECs	2, 4, 8, 16, 32 million/kg	IV	<24 h	(39)

*IV, intravenous; IT, intrathecal; IA, intra-arterial; IC, intracrainial; ICb, intracerebral; IN, intranasal*.

Multiple factors influence the efficacy of cell transplantation and treatment outcomes, and these considerations may be specific to the cell type. Therefore, thorough investigation must be undertaken in order to develop the most effective and safest combination of cell type, dosage, route of administration and timing of delivery (10, 64). Stroke type and hence infarct size and location need to be considered to enable targeted treatment. The choice of cell type and delivery method will enable the homing of the cells to the site of injury and the level of efficacy that can be achieved. Furthermore, there are additional considerations if the cell therapy is administered to treat a subacute or a chronic stroke since the blood brain barrier will be less permeable compared to the acute stages of stroke (65). In these instances, the cell delivery would have to be intrathecal. This approach is more invasive and would require the patient to stop anti-thrombotic medication, thereby risking the recurrence of recurrence during this period.

In addition to the clinical challenges to applying a cell therapy for stroke, there are the manufacturing challenges. There are limited enabling technologies for manufacturing cells at a commercial scale (33). This process must be developed for each cell type, and optimized for the production of a high yielding, quality product. Development of cell manufacturing processes is exhaustive, expensive, and time-consuming. Even when the processes is optimized, the use of autologous BMSCs can be limited by the fact that they require *in vitro* expansion for a week or longer to obtain a therapeutic dose of cells (53). This eliminates the possibility of autologous treatment within a few hours of stroke onset. This might be the reason that most of the active trials to date have focused on allogeneic cell therapies as detailed in Table 2. Many cell lines are additionally immortalized and/or otherwise modified (e.g., MASTERS trial, ACTIsSIMA trial; see section Importance of Standardization in Outcome Reporting). This eliminates the variability in yield and potency as would be the case with autologous cell lines and is amenable to a streamlined production process with predetermined product quality and yield.

For successful trials reaching a phase where patients will be recruited at multiple sites on an international scale another challenge arises. The regulatory frameworks and authorities differ between countries. For example in the US clinical trials are regulated by the Food and Drug Administration (FDA) whereas in Australia Therapeutic Goods Administration (TGA) and in the UK it is The Medicines and Healthcare Products Regulatory Agency (MHRA). Their authorities, tasks and processing times differ from country to country. In an attempt to support the planning and implementation of international clinical research the NIH offers an online database, ClinRegs (https://clinregs.niaid.nih.gov), which compares the country-specific research regulatory information between 20 different countries (e.g., US, Canada, UK, China, India, Australia, and South Africa). Commonly discussed challenges for sponsors of multiregional trials are planning and trial design, data recording and analysis (statistics), clinical (medical standards of care, access to care, and qualification of personel), regulatory operational, and ethical practices (66). Important points to consider such as differences in patient populations, efficacy, clinical investigator sites was recently summarized by Shenoy (67). The review gives examples with a focus on China and the United States.

In current agreement trials follow the principles of Good Clinical Practice (GCP). These have their origin in the World Medical Association's Declaration of Helsinki, 1964 and were used as a basis for the guidelines published by the International Council for Harmonization (www.ich.org) in 1996. These guidelines have been adopted by several regulatory agencies from different countries. They are recently being updated (67).

## Cell Therapy Clinical Trials for Stroke

Several reports which have set out to analyze the outcomes of different cell therapy clinical trials for stroke have pointed out the risk for biases (3, 68, 69). The most commonly found was attrition bias (incomplete outcome data), reporting bias (selective reporting of results), and selection bias (random sequence generation, allocation concealment bias). There is, therefore, a need for updated guidelines and the implementation of standardization of recording and reporting data from cell therapy trials for stroke and likely, other conditions. The formation of clinical trial networks attempts to address some of the challenges of running a clinical trial. It offers support with trial coordination in general, site and data management, statistical analysis, patient recruitment in particular (70). This may especially benefit investigator-initiated trials where the trial team has limited experience with cell-based therapies.

To date, several pre-clinical and clinical trials indicate that cell-based therapies are generally safe, however the mechanisms through which the cells exert their therapeutic efficacy requires further investigation (25, 35, 36, 38, 44, 52, 53, 71). Agreeable safety profiles with functional improvements in patients with stroke have been reported for example after transplantation of neuronal cells differentiated from a teratocarcinoma cell line (24), immortalized human neural stem cell (38), transformed allogeneic BMSCs (44), and autologous BMSCs (72). The guidelines on the development of cell therapies for stroke, Stem Cell as an Emerging Paradigm in Stroke (STEPS) (73–75) (see Chapter 4 for more details on STEPS) outlined the need for long-term safety testing when the cells used are highly proliferative and easily differentiate. In a follow-up study from a trial published in 2005 (76). Lee et al. (71) analyzed long-term safety in an open-label, placebo controlled trial with 85 patients who suffered from ischemic stroke within the last 90 days (both trials have no NCT number). 5 × 10^7^ autologous BMSCs were administered intravenously twice; 4 and 6 weeks after bone marrow aspiration. Patients were followed up for 5 years, and it was found that the SC transplant was safe. Another trial by Fang et al. (77) followed up their patients for 4 years (NCT01468064). This trial was a two-center, randomized, placebo-controlled phase I/IIa trial treating 18 patients suffering from acute cerebral infarct within 7 days of stroke onset. 5 × 10^6^ cells/kg body weight BMSCs or endothelial progenitor cells (EPCs) were administered intravenously in 2 doses 4 and 5 weeks after bone marrow aspiration. This study also found that the treatment was safe. Long-term studies are currently still the exception, and more data is needed to understand if safety can be guaranteed for every cell type used in a potential therapy.

Most results reported up to date, demonstrate safety, but do not show sufficient data for clinical efficacy. The trial mentioned above by Fang et al. (77) for example reported no significant improvement in functional outcome. Recently, adjunctive therapies have been discussed to be a way to tackle low efficacy. The combination of a stem cell therapy with a drug that is able to improve neurogenesis and angiogenesis and/or reduce inflammation and hence working along the same pathways as stem cells do, could amplify efficacy. Several drugs with such biological activity have been already identified (78). For example, for the treatment of stroke G-CSF (granulocyte-colony stimulating factor) has been proposed as an adjunct therapy to stem cell treatment of human umbilical cord blood cells (79). Further preclinical studies are required for the translation of combinatorial therapies into the clinic.

A successful stem cell therapy for stroke must be safe, effective, applicable to a broad spectrum of stroke patients, and economically viable. Current trials differ in cell type, route, dose, and time of administration, as well as patient recruitment criteria. However, independent of the heterogeneity between trials, the most noteworthy adverse events such as seizures, headaches, and administration procedure-related events have been similar.

Patient selection is a critical component in reducing heterogeneity within any given trial cohort, however, this is particularly the case for stroke which has a heterogeneous clinical presentation. As such, an investigator may wish to exclude patients with certain comorbidities, and this should be incorporated into the trial design in order to limit heterogeneity and more accurately report on efficacy. Given that biological markers of stroke recovery are currently unavailable, the methods for ascertaining clinical improvement must be carefully chosen in order to provide meaningful data. The following section describes three industry-sponsored and two investigator-initiated cell therapy clinical trials as examples of trial design, execution and evaluation.

### MASTERS and Treasure Trials

The Athersys Inc. funded MASTERS clinical trial (NCT01436487) was a phase 2, randomized, double-blinded, placebo controlled, dose-escalation trial, using allogeneic, bone marrow-derived, multipotent adult progenitor cells (MultiStem) (52). The MASTERS trial concluded in 2015, after treating 126 patients (134 enrolled) diagnosed with moderate to moderate-severe ischemic stroke (53). Patients were divided into three treatment groups: 1. Treatment 24–36 h after stroke onset with 400 million cells or placebo, 2. Treatment 24–36 h after stroke onset with 1.2 billion cells or placebo, and 3. Treatment 24–48 h after stroke onset with 1.2 billion cells or placebo. Sixty-five patients received cells and 61 patients received the placebo. No dose-limiting toxicity events or treatment-emergent adverse events (TEAE) were recorded. The investigators concluded that intravenously administered MultiStem was safe and well-tolerated, even at the higher dose. While changes in pro-inflammatory cytokines were noted, there was significant clinical improvement (at 90 days: mRS ≤ 2, Barthel Index ≥95, NIHSS ≥75% improvement). A major learning from the MASTERS trial is perhaps that of the logistics around cell manufacturing and provision of a living biologic within a relatively short treatment window. The MASTERS trial was initially designed with treatment within 24–36 h. However, the investigators ultimately changed their protocol to include a cohort at 24–48 h due to the logistical challenges in delivering cell products within the original timeframe (53). Upon conclusion of the MASTERS trial, the investigators concluded that the timing of the treatment is absolutely crucial, such that clinical efficacy was lost within the 36–48 h time window, thereby supporting an earlier intervention (53).

Indeed, this concept of an earlier intervention is currently being investigated by Athersys in the MASTERS-2 (NCT03545607) trial which commenced recruitment in 2018. The MASTERS-2 trial is a phase 3 quadruple-blind, randomized control trial to study the safety and efficacy of MultiStem, in patients suffering from acute ischemic stroke. The treatment (1.2 billion cells) are administered intravenously within 18–36 h of stroke onset. In addition to the MASTERS-2 trial, a different sponsor, Helios, is currently running a placebo controlled, multicentre phase 2/3 trial, TREASURE (NCT02961504) (80) where patients are recruited exclusively from Japan. The TREASURE trial also administers the cells intravenously within 18–36 h of stroke onset where MultiStem is administered at the same dose of 1.2 billion. Both trials are currently recruiting as of the preparation of this review and are estimated to conclude in 2020.

### ACTIsSIMA Trial

The ACTIsSIMA trial is a SanBio sponsored Phase 2 double-blinded, sham-surgery controlled trial using allogeneic BMSCs transfected with a plasmid coding for a Notch I domain (SB623, NCT02448641). The SB623 cells are administered via stereotactic, intracranial injection to eligible patients suffering from chronic ischemic stroke. Preclinical studies indicate that these gene-edited allogeneic BMSCs surpassed the outcome of unmanipulated BMSCs in rodent stroke models (44) and were tested in a phase I/II dose escalation trial (NCT01287936) (36, 81). The open-label phase 1/2a safety trial enrolled 18 patients having suffered from a subcortical stroke within the past 6–60 months. Doses of 2.5, 5, or 10 million SB263 cells were administered via a stereotactic placement within the margin of the site of the infarct. The only TEAE recorded were related to the procedure, rather than the cells. Of the 18 recruited patients, 16 have completed the 12 months follow-up. Significant improvements were recorded in this study—European Stroke Scale: mean increase 6.88; National Institutes of Health Stroke Scale (NIHSS): mean decrease 2.00; Fugl-Meyer total score: mean increase 19.20; Fugl-Meyer motor function total score: mean increase 11.40. No changes on the modified Rankin Scale (mRS) were recorded (36, 51). Based on the conclusion that SB623 cells were safe and associated with an improvement in clinical outcome, the Phase 2 ACTIsSIMA trial commenced in 2016 where two cohorts of patients received either a dose of SB623 cells at 2.5 or 5 million cells, or a sham placebo will be randomized in a 1:1:1 ratio. The trial is expected to conclude in 2019.

### Pisces Trial

Another industry sponsored trial to show promise in the cell therapy space for stroke is the ReNeuron sponsored trial investigating the potential of genetically modified human fetal cortical neuroepithelial cells for stroke (82). This cell therapy product is genetically modified using a retro-viral insertion of the modified growth factor c-mycERTAM (CTX0E03 DP) which overcomes the manufacturing problem of slow growing MSC through the transient expression of c-myc using a tamoxifen-estrogen receptor system (38, 81). The Pilot Investigation of Stem Cells in Stroke Trial (PISCES) was a Phase 1/2 open-label, dose-escalation safety trial (NCT01151124). The trial was based on preclinical studies in rats. Specifically, the injection of CTX0E03 DP was tested in a rat model of stroke induced by middle cerebral artery obstruction, where 450,000, 45,000 or 4,500 CTX0E03 DP were injected 4 weeks after MCOA (middle cerebral artery occlusion). Functional outcomes were assessed 2 weeks after cell implantation. Notably, significant functional improvement was only noted at the highest dose (41) and only when cells were delivered via an intraparenchymal injection, but not when delivered via intracerebroventricular injection which failed to achieve graft survival or functional improvement (42). Positive outcomes were associated with endogenous neurogenesis (83) and angiogenesis (84).

In PISCES, 11 patients suffering from ischemic stroke received a stereotactic ipsilateral putamen injection of CTX0E03 DP 6–60 months after stroke onset. Doses of 2, 5, 10, or 20 million cells were administered. As was the case for the MASTERS trial, the investigators struggled with the logistics of cell therapies and were able to treat only two patients at the highest dose. Safety was assessed over a 2-year period and no TEAE were recorded. Overall, the investigators concluded that this cell therapy is feasible and safe. Following this study PISCES II was launched in 2014 (NCT02117635). PISCES II is a phase 2a, open-label, safety trial where 20 million CTX0E03 DP cells were administered intracranially via stereotaxic neurosurgery. As of the preparation of this review, 23 patients have been recruited to PISCES II and received CTX0E03 DP cells. Results to date are available on the ReNeuron website (www.reneuron.com) but not compiled in a peer-reviewed publication. The 12-month follow-up showed an improvement in mRS and Barthel Index in 50 and 41% of enrolled patients, respectively. It was concluded that the treatment is safe and feasible, and a placebo-controlled, randomized phase 2b trial was started in 2018—PISCES III (NCT03629275)—where a larger number of patients will be recruited (110) and is estimated to conclude by late 2019. No further detailed analysis of the trial data is currently available.

### I-ACT Trial

The I-ACT trial is an investigator-initiated single site trial. I-ACT is a open-label, dose escalation safety phase 1 study (39). Patients will receive an intravenous infusion of 2, 4, 8, 16, and 32 million cells per kg body weight of allogeneic placenta-derived hAECs within 24 h of stroke onset. The study is based on preclinical data in several mouse and marmoset models of cerebral ischemia demonstrating neuroprotection and the facilitation of mechanisms of repair and recovery (85). The trial is estimated to conclude mid 2020 including a 1-year follow up.

### CoBIS2 Trial

The CoBIS2 trial is a continuation of CoBIS1. CoBIS1 was an investigator-initiated, multicentre, open-label, phase 1 safety study where 10 patients were recruited (37, 55, 86, 87). Allogeneic umbilical cord blood (UCB) containing 0.5–50 million total nucleated cells per kg bodyweight was administered intravenously 3–10 days post-stroke onset. Patients showed functional improvement 3 months past stroke onset. Fifty percent of patients showed improvement of one grade of mRS (mean mRS of 2.8 ± 0.9). NIHSS improved by at least 4 points (mean 5.9 ± 1.4). All patients demonstrated improvement in activities of daily living (Barthel Index mean increase 52.0 ± 24.7). The results conclude that the treatment is safe, feasible and suggestive of functional improvement. Based on these outcomes CoBIS2 was initiated. CoBIS2 is a multicenter, placebo controlled, randomized, double blinded, phase 2 study. CoBIS2 plans to recruit 100 participants. Doses of UCB and time frame of treatment remained the same. The trial is expected to conclude in 2020.

As can be seen from the trials above, the choice of route of administration is either intracranial or intravenous. And while some trials deliver a cell dosage based on bodyweight (I-ACT and CoBIS2), others deliver a fixed dose of cells. The completed trials preceding these current trials have provided clear results on treatment safety, but clinical efficacy remains uncertain. The MASTERS trial (52, 53), the InVeSt trial (44) and another phase 1 trial administering autologous BMSCs (88), did not report significant functional improvement. The PISCES trial however, linked improved neurological function in patients with chronic stroke to the treatment with genetically modified, immortalized human NSCs (38). This is certainly encouraging despite the absence of significant efficacy. Furthermore, the treatment in some cases (e.g., MASTERS trial) was linked with lower rates of mortality and TEAE (52).

## Importance of Standardization in Outcome Reporting

In order to assess the overall potential and combined outcomes of stem cell therapy for stroke, it is important to assess the outcomes across the various completed trials. The need for quality standards and documentation of study outcomes for pre-clinical and clinical stem cell research in stroke was identified a decade ago. In 2009, experts from academia and industry, members of the National Institute of Health (NIH) and the Food and Drug Administration (FDA) published their first meeting report, Stem Cell as an Emerging Paradigm in Stroke (STEPS) as a consensus-based guideline on the development of cell therapies for stroke, with a focus on the translation of pre-clinical studies and the design and conduct of early- and late-stage clinical trials for acute and chronic stroke (73). These guidelines have since been updated in 2011 (74) and 2014 (75).

Many of the problems identified in this exercise were also reported in several meta-analyses, in particular the lack of consistent reporting of safety and efficacy data for combinatorial (89) or mono-therapies (3), as well as heterogeneity in study design [e.g., single-arm (90)], a cell type [e.g., MSCs, (91)] or a stroke type [ischemic (69)]. Recently, Nagpal et al. (3) compared several different early-phase cell therapy trials while disregarding the cell type, treatment administration or study design differences. Overall, they concluded that the administration of different types of stem cells was feasible and safe. None of the adverse events reported could be ascertained to be related to the respective cell therapies. Nevertheless, additional research is still needed in order to demonstrate efficacy and enable market approval. As full recovery is unlikely, the outcome of any given stroke trial is dependent on the estimation of functional neurological improvement and structural recovery. Nagpal et al. drew conclusions based on changes to the Barthell index, the modified Rankin scale, and NIH Stroke Scale values across different trials, where despite indications of improvement, the magnitude of impact was small. Currently, it is impossible to draw conclusions with regard to optimal treatment protocols due to the limited data available from a small number of clinical studies comprised of small cohorts in a notoriously heterogeneous disease.

## Cost-to-Benefit of Cell Therapy Trials

**Figure 1:**
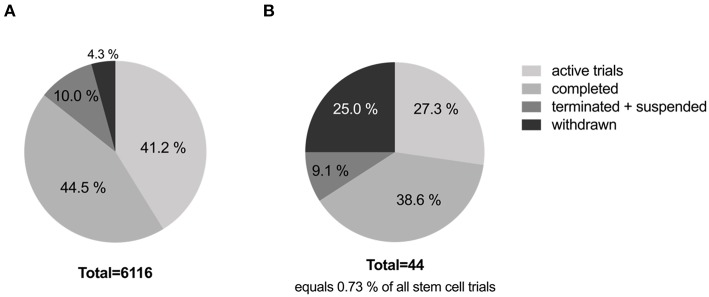
**(A)** Clinical trials-stem cells. **(B)** Clinical trials-stem cells + stroke.

More than 6,000 trials are registered with www.clinicaltrials.gov employing different types of stem cells, addressing different types of disorders and/or diseases (excluding unknown status trials). Currently, 41.2% of these stem cell clinical trials are active and 44.5% are completed. And of the registered stem cell trials, only 0.73% address stroke, with 38.6% being completed, and 27.3% currently active. Interestingly, 25% of stroke trials have been withdrawn and another 9.1% were terminated or suspended. See Figure 1 for details. Most trials were withdrawn or terminated due to low recruitment rates or lack or termination of funding. Strikingly, all completed trials are phase 1 and 2 trials, with only 1 in 5 trials moving on to the next phase. Of the 13 currently active trials only two trials have reached phase 3. These are the MASTERS-2 trial (Athersys Inc.) investigating a stem cell treatment for adults who have suffered an acute ischemic stroke, and the STARTING-2 trial (Samsung Medical Center, Korea) determining the efficacy of intravenous transplantation of autologous MSCs to treat acute ischemic stroke. On average clinical trials took 3.3 years from start to completion. From the data collected (see Table 2), one can estimate that a cell therapy for stroke will take >10 years to progress from phase 1 to phase 3 without considering any preceding preclinical research or process development.

Given this extremely long “gestation” for translating cell therapies for stroke, it is important to also consider the cost-to-benefit ratio. The US Department of Health & Human Services published a comprehensive report on clinical trial costs as part of their analysis of current barriers for drug development (92). The top three cost drivers of clinical trial expenditures were clinical procedure costs (15–22% of total), administrative staff costs (11–29% of total), and site monitoring costs (9–14% of total). Generally, costs increase with every trial phase: phase 1 on average being about US$ 4 million, phase 2 about US$ 13 million and phase 3 and 4 about US$ 20 million each. This totals to an average of US$ 57 million to take a therapeutic through its clinical trial stages. The costs of a trial depend largely on the therapeutic area being targeted. The most expensive clinical trials (phase 1–3) focus on pain and anesthesia US$ 71.3 million, ophthalmology US$ 49.8 million, and anti-infectives US$ 41.2 million. Trials on treatments focused on the CNS are estimated to cost US$ 37 million, and US$ 34.4 million on cardiovascular diseases. In order to understand if the costs of a trial are economically justifiable, one has to consider the financial costs that stroke has on a society, which was analyzed on an international level in 2004 (93). This study was done over a decade ago, and an update would certainly be necessary as the prevalence of stroke has continued to rise. In 2012, the total cost of stroke to Australia was estimated at AU$ 5 billion with health cost being AU$ 881 million and productivity cost being AU$ 3 billion (5). The costs of stroke to the UK health and social services in the same year were estimated at AU$ 5.2 billion (£ 2.9 billion) (94). Efficacious stroke interventions are expected to significantly reduce the financial costs. However, this does not take into consideration the costs of stem cell-based therapies. This is despite attempts to assess the cost and benefits of medical research since the 1990's (95), with analysis of funds going into specific areas of medical research (e.g., stroke, cancer, and dementia) (94). Information on the actual costs of clinical trials in general or even specific clinical trials is scarce.

In 2017 the Australian Government published an economic evaluation of 25 clinical trials to assess the overall health and economic impact of investigator-initiated clinical trials (70). This report only included independent investigator-initiated trials that were part of clinical trial networks in Australia, in phase 2 and beyond. Included in this report were 7 trials conducted within the Australasian Stroke Trials Network between 2004 and 2014. The combined costs of 4 trials was estimated to be AU$ 32 million (excluding early phase trials, pilot and feasibility trials and observation studies). The gross economic benefit of these trials was estimated at AU$ 327 million. Thus, the benefit for these late-phase trials was estimated to be AU$ 10 per AU$ 1 invested. The report estimated a benefit of AU$ 5 for every AU$ 1 invested with a total gross benefit of AU$ 2 billion for all 25 trials analyzed, with a cumulative reduction in health service costs in the order of AU$ 580 million. Since none of the trials included in the report used any kind of cell therapy, an estimation of the benefit of cell-therapy clinical trials could not be made. However, it is clear that clinical trials are quantifiably beneficial and while the analysis covered a decade's worth of trials, it did not include any data from the early stage trial counterparts. This further emphasizes the length of time required for clinical development of any new treatment for stroke before clinical benefit is seen, let alone to see profit.

In 2018, the costs of producing for autologous cell therapies have been estimated to be US$ 94 per million cells (96). For a dose of 2 million cells per kg, assuming that a patient weighs 70 kg, the costs would be US$ 13,160 per dose. The costs of drug development influences the pricing of any clinical therapy, but several factors can also contribute to the cost, such as market size, competing products, the quality-adjusted life year (QALY), and what the consumer is willing to pay (97, 98). At the moment, there is no approved stem cell therapy for stroke. Stem cell therapies approved by the FDA include cord blood and a small number of cell lines (e.g., modified T-cells, chondrocytes, and fibroblasts). There are three different approved stem cell therapies on the market. The European commission approved a stem cell product, Alofisel (Takeda) in 2018, however FDA approval is still pending at the time of the writing of this review. Alofisel is an ASC treatment for perineal fistulas in patients with Crohn's disease. One course of treatment is US$ 61,000 (99). Another approved product is Holoclar (Holostem Terapie Avanzante), which was the first stem cell therapy to receive market authorization in the EU. Holoclar is comprised of corneal epithelial cells used to treat chemical burns of the eye, costing US$ 102,000 (99). TEMCELL (JCR Pharmaceuticals) is approved in Japan for the use of BMSCs to treat Graft vs. Host Disease, costing about US$ 170,000 per course (100). The price tags on these approved therapies can only hint at expected costs of cell therapies for stroke. Furthermore, every country has their own health care board deciding if a treatment would be covered by the national health care system, thus making it impossible to estimate the actual out-of-pocket costs a patient would incur. It is evident that a treatment which improves functional outcomes in stroke, as can be seen from other recent interventions, will have a significant benefit on health costs (5).

## The Role of Industry-Led Research in the Development of Cell Therapies for Stroke

When assessing the success of clinical trials there is always the question if industry-led research is more successful than academic research. This has been critically analyzed and reviewed over the last decade as scientists and public are aware of the inherent risk of biases in industry funded studies (101–104). Recently, Lundh et al. (105) compared studies with and without industry funding from 75 papers on reported efficacy, conclusions and risk of bias. The papers selected were on primary research studies, empirical studies and randomized clinical trials (58 papers) focusing on drug and device development, but without a focus on a specific disease or type of therapy. Industry funded projects were found to demonstrate favorable efficacy toward tested treatments, with less substantive conclusions. Over 60% of published findings from cell therapy clinical trials reported positive outcomes, with a trend toward a higher proportion of positive reports from industry funded trials (106). Expectedly, the more complete and detailed studies were published in higher impact factor journals where data are presumably subjected to a higher level of scrutiny.

Reporting according to CONSORT (107) or also SPIRIT (Standard Protocol Items: Recommendations for Interventional Trials) (108) identifies funding sources and possible conflicts of interest but are not without limits compared to more recent recommendations by Hakoum et al. (109) that specifically pay address the characteristics of funding of clinical trials. External influences exerted on research and clinical trials are difficult to trace and often remain unclear when the results are published at the end of a trial (104). Currently about 28.5% of all active, interventional clinical trials registered with www.clinicaltrials.gov are industry funded. In relation to current active, interventional trials focused on stem cell therapies for stroke, 66.7% are industry funded (Table 2). Late-stage trials (phase 2 and 3) are predominantly industry funded (58.3%) whereas early-stage trials (phase 1 and 1/2) are funded by other means (62.5%). This might be due to the fact that the later phases of clinical testing incur larger costs that are beyond the funding quanta of philanthropic or government bodies, or that promising late-stage trials may be of greater interest to industry.

## Conclusion

It is clear that there are challenges in the use of cell therapies for stroke (Table 1) that remain to be addressed in the future. A major issue certainly is the need for standardized outcome reporting that is free of bias and enables comparison of different trials. Furthermore, optimized and more efficient bioprocesses need to be urgently developed to reduce the cost of production and in doing so, treatment costs. Most studies showed safety and feasibility for cell therapy for stroke independent of cell type and route of administration. However, there remains limited proof of efficacy. We and others will be watching closely for the outcomes of current stroke clinical trials utilizing cell therapies, as we await the evidence for clinical efficacy and impactful functional improvement that is desperately needed to spur this field ahead.

## Author Contributions

MK and RL drafted the manuscript. MK prepared all figures and tables. All authors provided input according to their expertise and discussed the manuscript.

### Conflict of Interest Statement

The authors declare that the research was conducted in the absence of any commercial or financial relationships that could be construed as a potential conflict of interest.
